# Evaluation of the Alinity m Resp-4-Plex Assay for the Detection of Severe Acute Respiratory Syndrome Coronavirus 2, Influenza A Virus, Influenza B Virus, and Respiratory Syncytial Virus

**DOI:** 10.1128/spectrum.01090-21

**Published:** 2022-02-02

**Authors:** Wei Zhen, Ryhana Manji, Elizabeth Smith, Jeffrey Wuitschick, Danijela Lucic, Gregory J. Berry

**Affiliations:** a Infectious Disease Diagnostics, Northwell Health Laboratories, Lake Success, New York, USA; b Abbott Molecular, Des Plaines, Illinois, USA; c Department of Pathology and Laboratory Medicine, The Donald and Barbara Zucker School of Medicine at Hofstra/Northwell, Uniondale, New York, USA; Labcorp

**Keywords:** nucleic acid amplification test, multiplex molecular assays, accurate diagnosis, sample-to-answer platform, respiratory virus detection, SARS-CoV-2

## Abstract

The rapid emergence of the coronavirus disease 2019 (COVID-19) pandemic has introduced a new challenge in diagnosing and differentiating respiratory infections. Accurate diagnosis of respiratory infections, including severe acute respiratory syndrome coronavirus 2 (SARS-CoV-2), is complicated by overlapping symptomology, and stepwise approaches to testing for each infection would lead to increased reagent usage and cost, as well as delays in clinical interventions. To avoid these issues, multiplex molecular assays have been developed to differentiate between respiratory viruses in a single test to meet clinical diagnostic needs. To evaluate the analytical performance of the FDA emergency use authorization (EUA)-approved Abbott Alinity m resp-4-plex assay (Alinity m) in testing for SARS-CoV-2, influenza A virus, influenza B virus, and respiratory syncytial virus (RSV), we compared its performance to those of both the EUA-approved Cepheid Xpert Xpress SARS-CoV-2, influenza A/B virus, and RSV assay (Xpert Xpress) and the EUA-approved Roche Cobas SARS-CoV-2 and influenza A/B virus assay (Cobas) in a single-center retrospective analysis. High concordance was observed among all three assays, with kappa statistics showing an almost perfect agreement (>0.90). The limit of detection (LOD) results for SARS-CoV-2 showed the Alinity m exhibiting the lowest LOD at 26 copies/mL, followed by the Cobas at 58 copies/mL and the Xpert Xpress at 83 copies/mL, with LOD results for the influenza A virus, influenza B virus, and RSV viral targets also showing equivalent or better performance on the Alinity m compared to the other two platforms. The Alinity m can be used as a high-volume testing platform for SARS-CoV-2, influenza A virus, influenza B virus, and RSV and exhibits analytical performance comparable to those of both the Xpert Xpress and Cobas assays.

**IMPORTANCE** The rapid emergence of SARS-CoV-2 has introduced a new challenge in diagnosing and differentiating respiratory infections, especially considering the overlapping symptomology of many of these infections and differences in clinical interventions depending on the pathogen identified. To avoid these issues, multiplex molecular assays like the one described in this article need to be developed to differentiate between the most common respiratory pathogens in a single test and most effectively meet clinical diagnostic needs.

## INTRODUCTION

Rapid, accurate diagnosis of severe acute respiratory syndrome coronavirus 2 (SARS-CoV-2) infection using nucleic acid amplification tests (NAATs) is essential for mitigating the spread of coronavirus disease 2019 (COVID-19), initiating timely contact tracing, and making treatment decisions (1, 2). COVID-19 cases continue to occur across the globe and soon will once again coincide with the traditional respiratory virus season (3, 4).

The concomitant presentation of SARS-CoV-2 with other common viral respiratory illnesses has introduced a new diagnostic challenge for clinical microbiology laboratories. In addition, the symptomologies of other “flu season” respiratory viral infections, such as influenza and respiratory syncytial virus (RSV), overlap that of COVID-19, making a definitive diagnosis based on clinical presentation alone impossible. Therefore, diagnostic testing must be performed for SARS-CoV-2 in addition to other circulating respiratory viruses to determine the cause of infection. However, the necessity to run multiple tests for each suspect patient can delay diagnosis and contact tracing, as well as initiation of appropriate treatment (5, 6). One solution to this issue has been the development of multiplexed molecular assays targeting SARS-CoV-2 and the other primary respiratory pathogens of concern during the respiratory virus season, such as influenza A virus, influenza B virus, and RSV. Subsequently, several commercially available multiplex molecular assays have been developed and have obtained FDA emergency use authorization (EUA), including the Alinity m resp-4-plex assay (Alinity m) (Abbott Laboratories, Chicago, IL, USA). The Alinity m resp-4-plex assay is a multiplex molecular assay for the qualitative detection and differentiation of RNA from SARS-CoV-2, influenza A virus, influenza B virus, and RSV in nasopharyngeal (NP) specimens and nasal specimens (7) and is performed on the automated Alinity m system. This sample-to-answer system performs sample preparation, reverse transcription PCR (RT-PCR) assembly, amplification, detection, and result analysis (8). An unrelated RNA sequence is also spiked into the sample reaction mixture to serve as an internal control. The Alinity m system is an automated, high-throughput, sample-to-answer, random-access molecular platform (8) and uses real-time PCR and ReadiFlex technology, allowing the system to run statum (STAT) samples and process 300 samples in approximately 8 h, with a time to first result of <2 h. The system also has an amplification reagent capacity of 20 reagent packs that can be stored onboard for 4 days.

In this study, the analytical performance and sensitivity of the Alinity m resp-4-plex assay were compared to those of two other emergency use authorization-approved multiplex molecular assays, the Xpert Xpress SARS-CoV-2, influenza A/B virus, and RSV assay (Cepheid, Inc., Sunnyvale, CA) and the Cobas SARS-CoV-2 and influenza A/B virus assay (Roche Diagnostics Corporation, Indianapolis, IN).

## RESULTS

The comparative performance of the Alinity m, Xpert Xpress, and Cobas assays for the detection of SARS-CoV-2, influenza A virus, influenza B virus, and/or RSV was assessed. A high degree of agreement was observed, with kappa statistics showing an almost perfect agreement (>0.90) with the reference standard result for all three assays (Table 1).

**TABLE 1:** Performance comparison of three FDA EUA assays for SARS-CoV-2, influenza A virus, influenza B virus, and/or RSV to the reference standards^*a*^

Molecular assay	Analyte	Result	No. of specimens with result in original standard-of-care testing (*n* = 20 samples/analyte + 20 samples negative for any analyte)^*b*^		Value (95% CI) for^*c*^:		
			Detected	Not detected	Kappa^*d*^	PPA	NPA
Xpert Xpress SARS-CoV-2, flu A/B, and RSV	Influenza A virus^*e*^	Detected	19	0	1.00 (1.0–1.0)	100 (0.82–1)	100 (0.95–1)
		Not detected	0	80			
	Influenza B virus	Detected	19	0	0.969 (0.91–1.0)	95 (0.76–0.99)	100 (0.95–1)
		Not detected	1	79			
	SARS-CoV-2	Detected	20	0	1.00 (1.0–1.0)	100 (0.83–1)	100 (0.95–1)
		Not detected	0	79			
	RSV^*f*^	Detected	19	0	0.969 (0.91–1.0)	95 (0.76–0.99)	100 (0.95–1)
		Not detected	1	79			

Cobas Liat SARS-CoV-2 and flu A/B	Influenza A virus	Detected	19	0	1.00 (1.0–1.0)	100 (0.82–1)	100 (0.95–1)
		Not detected	0	80			
	Influenza B virus	Detected	19	0	0.969 (0.91–1.0)	95 (0.76–0.99)	100 (0.95–1)
		Not detected	1	79			
	SARS-CoV-2	Detected	19	0	0.969 (0.91–1.0)	95 (0.76–0.99)	100 (0.95–1)
		Not detected	1	79			

Alinity resp-4-plex assay	Influenza A virus	Detected	19	0	1.00 (1.0–1.0)	100 (0.82–1)	100 (0.95–1)
		Not detected	0	80			
	Influenza B virus	Detected	19	0	0.969 (0.91–1.0)	95 (0.76–0.99)	100 (0.95–1)
		Not detected	1	79			
	SARS-CoV-2	Detected	19	0	0.969 (0.91–1.0)	95 (0.76–0.99)	100 (0.95–1)
		Not detected	1	79			
	RSV^*f*^	Detected	20	0	1.00 (1.0–1.0)	100 (0.83–1)	100 (0.95–1)
		Not detected	0	79			

a The consensus result was defined as the result obtained from the original standard-of-care testing.

b One hundred NP specimens were selected for this validation based on previous standard-of-care results, among which 20 specimens each were initially singly positive for SARS-CoV-2, influenza A virus, influenza B virus, or RSV and 20 were negative specimens.

c CI, confidence interval; PPA, positive percent agreement; NPA, negative percent agreement.

d Agreement shown by kappa statistic: >0.90, almost perfect; 0.80 to 0.90, strong; 0.60 to 0.79, moderate; 0.40 to 0.59, weak; 0.21 to 0.39, minimal; 0 to 0.20, none.

e One influenza A virus-positive specimen was removed from the analysis due to insufficient volume for comparator testing.

f One influenza B virus-positive specimen selected from standard-of-care testing results was found to be RSV positive by the two molecular assays being evaluated. RSV was not included in the data analysis.

When we compared the overall analytical performance of the Alinity m to the reference standard results, we observed 100% positive percent agreement (PPA) for influenza A virus and RSV, 95% PPA for influenza B virus and SARS-CoV-2, and 100% negative percent agreement (NPA) for all four analytes. The Cobas assay exhibited 100% PPA for influenza A virus and 95% PPA for influenza B virus and SARS-CoV-2, and also observed 100% NPA for influenza A virus, influenza B virus, and SARS-CoV-2, with RSV not included on the panel. For the Xpert Xpress, we observed 100% PPA for influenza A virus and SARS-CoV-2, 95% PPA for influenza B virus and RSV, and 100% NPA for influenza A virus, influenza B virus, SARS-CoV-2, and RSV. One sample was detected as positive for RSV on the Alinity m, but RSV was reported as not detected by the Xpert Xpress; of note, the cycle number (CN) value from the Alinity was 39.31, indicating a low-viral-load specimen.

We also examined the limit of detection (LOD) for each of the three assays using intact, quantified viral particles and/or quantified simulated RNA virus. For SARS-CoV-2, the Alinity m exhibited the lowest LOD at 26 ± 21 copies/mL (mean ± standard deviation), followed by the Cobas at 58 ± 18 copies/mL and the Xpert Xpress at 83 ± 24 copies/mL. For influenza A virus, the Xpert Xpress had the lowest LOD at 32 ± 24 copies/mL, followed by the Alinity m at 36 ± 22 copies/mL and the Cobas at 77 ± 19 copies/mL. For influenza B virus, the Alinity m had the lowest LOD at 22 ± 22 copies/mL, followed by the Xpert Xpress at 38 ± 22 copies/mL and the Cobas at 122 ± 18 copies/mL. Finally for RSV, which is not included in the Cobas assay, the Alinity m had an LOD of 22 ± 23 copies/mL, while the Xpert Xpress had an LOD of 326 ± 14 copies/mL (Table 2).

**TABLE 2:** Summary of limit of detection results

Molecular assay	Analyte	No. of positive replicates at indicated dilution/total no. of replicates (copies/mL) (% positive)						LOD (copies/mL) (95% CI)^*a*^
		400	200	100	50	25	12.5	
Xpert Xpress SARS-CoV-2/flu A/B & RSV	SARS-CoV-2	5/5 (100)	10/10 (100)	10/10 (100)	8/10 (80)	4/5 (80)	NA^*b*^	83 ± 24
	Influenza A virus	5/5 (100)	10/10 (100)	10/10 (100)	10/10 (100)	5/5 (100)	5/10 (50)	32 ± 24
	Influenza B virus	5/5 (100)	10/10 (100)	10/10 (100)	10/10 (100)	5/5 (100)	2/10 (20)	38 ± 22
	RSV	5/5 (100)	5/10 (50)	0/10 (0)	0/10 (0)	0/5 (0)	NA	326 ± 14

Cobas Liat SARS-CoV-2/flu A/B	SARS-CoV-2	5/5 (100)	10/10 (100)	10/10 (100)	10/10 (100)	3/5 (60)	NA	58 ± 18
	Influenza A virus	5/5 (100)	10/10 (100)	10/10 (100)	9/10 (90)	3/5 (60)	2/10 (20)	77 ± 19
	Influenza B virus	5/5 (100)	10/10 (100)	9/10 (90)	7/10 (70)	5/5 (100)	4/10 (40)	122 ± 18

Alinity resp-4-plex assay SARS-CoV-2/flu A/B & RSV	SARS-CoV-2	5/5 (100)	10/10 (100)	10/10 (100)	10/10 (100)	5/5 (100)	7/10 (70)	26 ± 21
	Influenza A virus	5/5 (100)	10/10 (100)	10/10 (100)	10/10 (100)	5/5 (100)	3/10 (30)	36 ± 22
	Influenza B virus	5/5 (100)	10/10 (100)	10/10 (100)	10/10 (100)	5/5 (100)	9/10 (90)	22 ± 22
	RSV	5/5 (100)	10/10 (100)	10/10 (100)	10/10 (100)	5/5 (100)	6/10 (60)	22 ± 23

a The LOD was determined by Probit analysis.

b NA, not applicable.

## DISCUSSION

We evaluated the performance and analytical sensitivity of the Alinity m resp-4-plex assay for the detection of SARS-CoV-2, influenza A virus, influenza B virus, and/or RSV in comparison to two point-of-care, sample-to-answer, EUA multiplex assays, the Xpert Xpress and the Cobas. The results demonstrate that the three assays have high concordances, with kappa statistics showing an almost perfect agreement (>0.90) and PPA and NPA falling between 95% and 100% for each of the targets. In addition, the results of the LOD study demonstrate that the Alinity m assay had the lowest LODs of the three platforms for the SARS-CoV-2 and influenza B virus targets, with the Xpert Xpress showing an LOD for the influenza A virus (32 ± 24 copies/mL) equivalent to that found with the Alinity (36 ± 22 copies/mL). When comparing the LOD for RSV, the Alinity m assay had an LOD of 22 ± 23 copies/mL, which was substantially lower than the LOD for the Xpert Xpress of 326 ± 14 copies/mL. Of note, the Cobas assay does not target RSV as an analyte, so it was not included in the RSV analysis. Overall, the analytical performances of all three platforms were similar.

The main difference between the Alinity m and the other two platforms is that the Alinity m platform is an automated, high-throughput (900 tests per 24-h time period) test system that allows rapid testing of large numbers of samples. This feature is especially useful in the clinical microbiology laboratory setting during respiratory season, when large numbers of specimens can arrive at the same time and require a quick turnaround time. In contrast, the Xpert Xpress and Cobas assays are designed for near-patient testing and lower testing volumes. For example, while the Alinity m system can test 300 samples in an ∼8-h time period with a <2-h time to first result, the Xpert Xpress on the GeneXpert point-of-care testing system can test four samples at a time and the Cobas on the Liat system can test one sample at a time. This difference in testing volume should be considered in the context of the clinical testing needs (i.e., point-of-care testing versus higher-volume laboratory-based testing).

Overall, replacement of singleplex molecular assays with multiplex molecular assays has become a trend in clinical microbiology laboratories. This is especially true with respiratory testing, where it is impossible to determine which pathogen is causing disease based on symptomology alone. During respiratory season, it will be necessary to determine whether influenza virus, RSV, or SARS-CoV-2 is causing respiratory symptoms, so multiplex assays will fill this clinical need. While this multiplex approach is clearly more efficient and convenient, it is also important to note that the comparative LOD study also showed that sensitivity was not compromised by this approach.

One additional point to consider is that both the Alinity m and Xpert Xpress assays detect RSV, which is considered a significant respiratory pathogen in younger and elderly populations and those who are immunocompromised (9). This additional RSV viral target gives these two assays an advantage over the Cobas assay, especially when these groups are the predominant patient populations being tested.

In summary, this comparison demonstrates that the Alinity m resp-4-plex assay is a sensitive and accurate test for the simultaneous detection of SARS-CoV-2, influenza A virus, influenza B virus, and RSV, and this assay also has the capacity for large-scale testing. This high-volume and accurate testing ability can distinguish between SARS-CoV-2 and other respiratory viruses and help direct appropriate care for patients.

## MATERIALS AND METHODS

### Clinical samples.

Remnant NP specimens collected in 3 mL of universal transport medium (UTM; various manufacturers) were obtained after routine clinical testing at Northwell Health Laboratories, Lake Success, NY. A total of 100 NP specimens were selected for this validation based on previous standard-of-care results, among which 20 specimens each that were initially singly positive for SARS-CoV-2, influenza A virus, influenza B virus, or RSV and 20 negative specimens were collected from testing with the Panther Aptima EUA SARS-CoV-2 assay (Hologic, Inc., San Diego, CA), the GenMark ePlex respiratory pathogen panel (Roche Diagnostics, Carlsbad, CA), or the Luminex ARIES flu A/B & RSV assay (Luminex Corp., Austin, TX) (10, 11). The samples were stored at −80°C prior to testing with the Alinity m (7) and comparator assays. Once thawed, the samples were deidentified and aliquoted for testing with the Alinity m, Xpert Xpress, and Cobas assays. One influenza A virus-positive specimen was removed from the analysis due to insufficient volume for comparator testing. In addition, one influenza B virus specimen selected from standard of care was found to be RSV positive by all three molecular assays being evaluated, but RSV was not included in the data analysis due to not being detected by the reference standard.

This work was conducted as a quality improvement activity for assay validation purposes to complete clinical validation using deidentified remnant specimens and was exempt from institutional review board approval.

### Molecular comparator assays.

The Xpert Xpress assay (12) is a multiplex RT-PCR assay that differentiates between SARS-CoV-2, influenza A virus, influenza B virus, and RSV in NP or nasal samples and is run on the GeneXpert Xpress system. The Cobas assay (13) is an automated multiplex RT-PCR assay that differentiates between SARS-CoV-2, influenza A virus, and influenza B virus in nasal or NP swabs, is run on the point-of-care Cobas Liat system with results available in 20 min, and does not contain RSV as a target.

Individual assay and platform characteristics obtained from each package insert (7, 12, 13) are further described in Table 3.

**TABLE 3:** Basic performance characteristics and workflow evaluation for three EUA multiplex molecular assays

Parameter	Name or value		
manufacturer	Cepheid	Roche	Abbott
Assay	Xpert Xpress SARS-CoV-2, influenza A/B virus and RSV	Cobas SARS-CoV-2 and influenza A/B virus	Alinity m resp-4-plex
Detection platform	GeneXpert	Liat	Alinity m
Gene target(s) for^*a*^:			
SARS-CoV-2	E, N2	ORF 1a/b, N	RdRp, N
Influenza A virus	Matrix, PB2, PA	Matrix	Matrix
Influenza B virus	Matrix, nonstructural protein	Nonstructural	Nonstructural 1
RSV	Nucleocapsid	NA	Matrix
Sample types	Nasopharyngeal and nasal swabs and nasal wash/aspirate	Nasopharyngeal and nasal swabs	Nasopharyngeal and anterior nasal swabs
Sample vol required (µL)	300	200	500
TCID_50_/mL (unit of measure)^*b*^			
Influenza A virus (TCID_50_/mL)	0.004/0.087	0.02/0.002	0.002/0.015
Influenza B virus (TCID_50_/mL)	0.04	0.002/0.004	0.02/0.05
RSV (TCID_50_/mL)	0.43/0.22	NA	0.3/0.1
SARS-CoV-2 (as indicated)	131 copies/mL	0.01 TCID_50_/mL	0.005 TCID_50_/mL
Maximum throughput	4 per instrument (4-module configuration)	1 per instrument	300 samples in 8 h
Assay run time	36 min	20 min	∼120 min for first time to result

a E, envelope; N2, nucleocapsid gene region 2; ORF, open reading frame; RdRp, RNA-dependent RNA polymerase; PB2, polymerase basic protein 2 (polymerase subunit); PA, polymerase acidic protein (polymerase subunit); NA, not applicable.

b TCID_50_, 50% tissue culture infective dose.

### Analytical sensitivity (LOD).

The limit of detection (LOD) for each of the assays was assessed on each of the three evaluated platforms using the following two commercially available verification panels: for SARS-CoV-2 and RSV, serial dilutions of a quantified Accuplex SARS-CoV-2, influenza A/B, and RSV verification panel from SeraCare (catalog no. 0505-0183; SeraCare, Milford, MA), and for influenza A/B virus, serial dilutions of quantified influenza A virus (H1) and influenza B virus stock from ZeptoMetrix (catalog nos. NATFLUAH1-STQ and NATFLUB-STQ; Buffalo, NY). Universal virus transport medium from BD Biosciences (reference no. 220220; BD, Sparks, MD) was used as the diluent to prepare the following concentrations of each virus, with replicates ranging from 5 to 10 at each dilution: 400, 200, 100, 50, 25, and 12.5 genomic copies/mL. The LOD was determined as the lowest detectable dilution with a 95% probability of detection by Probit analysis.

### Statistical methods.

The analytical concordances of the Alinity m, Xpert Xpress, and Cobas assays were compared. The reference standards were established as the results obtained from the original standard-of-care testing on the Panther Aptima EUA SARS-CoV-2 assay, the GenMark ePlex respiratory pathogen panel, and the Luminex ARIES flu A/B & RSV assay. Positive percent agreement (PPA), negative percent agreement (NPA), kappa (κ), Probit analysis, and two-sided (upper/lower) 95% confidence intervals (CI) were calculated using Microsoft Office Excel 365 MSO software (Microsoft, Redmond, WA).
